# Outcomes of surgery and/or combination chemotherapy for extraskeletal osteosarcoma: a single-center retrospective study from China

**DOI:** 10.1038/s41598-019-41089-1

**Published:** 2019-03-18

**Authors:** Zhichao Liao, Minghan Qiu, Jilong Yang, Yun Yang, Lei Zhu, Bo Yang, Xu Bai, Peipei Xing, Jin Zhang, Ruwei Xing, Sheng Teng, Jun Zhao

**Affiliations:** 1 Departments of Bone and Soft Tissue Tumor, Tianjin Medical University Cancer Institute & Hospital, Tianjin, 300060 People’s Republic of China; 2 National Clinical Research Center of Cancer, Tianjin Medical University Cancer Institute & Hospital, Tianjin, 300060 People’s Republic of China; 3 Department of Oncology, Tianjin Union Medical Center, Tianjin, 300121 People’s Republic of China; 4 Departments of Molecular Imagine, Tianjin Medical University Cancer Institute & Hospital, Tianjin, 300060 People’s Republic of China; 5 Departments of Pathology, Tianjin Medical University Cancer Institute & Hospital, Tianjin, 300060 People’s Republic of China; 6 Departments of Radiation Oncology, Tianjin Medical University Cancer Institute & Hospital, Tianjin, 300060 People’s Republic of China

## Abstract

Extraskeletal osteosarcoma (ESOS) is an extremely rare malignancy with poor prognosis, accounting for 2–4% of all osteogenic sarcomas. The purpose of this study was to examine the oncological outcomes of this disease related to surgical treatment and/or combined adjuvant therapies and to analyze the associated prognostic factors in ESOS. From January 1990 to June 2016, 22 patients with primary ESOS were analyzed in this retrospective study. Overall survival (OS) and progression-free survival (PFS) rates were calculated by Kaplan-Meier methods and compared with log-rank test. 22 patients were diagnosed with ESOS, 19 showed localized diseases and 3 presented with metastatic lesions. The median age at diagnosis was 55.5 years. Surgery resection was performed for all patients, 18 of whom received adjuvant chemotherapy. The median follow-up time was 48.5 months. There were 10 cases of recurrence and 9 patients developed new metastases. The 5-year OS rate for all patients was 58%. For localized cohort, the 5-year OS rate was 62%, and the 3-year PFS rate was 31% with a median PFS of 16 months. Univariate analysis of related prognosis factors showed that larger size of tumor (>5.5 cm) and higher histologic grade emerged as significant factors associated with worse OS. The addition of combination chemotherapy has no effect found on OS or PFS in this study. In summary, for patients who presented with ESOS, larger tumor size and higher histologic grade indicate a lower OS rate. The combination chemotherapy does not improve the OS or PFS.

## Introduction

Extraskeletal osteosarcoma (ESOS) is an extremely rare malignant mesenchymal tumor that was first defined by Wilson in 1941, accounting for approximately 1% of all soft tissue sarcomas and 2–4% of all types of osteogenic sarcomas^1,2^. Unlike classic osteosarcoma, ESOS developed in patients with a mean age of 48 to 60 years commonly, often comes with a high probability of recurrence and metastasis after surgical treatment^2–4^. Due to the extremely low incidence of this disease, there are few literatures available, which were mainly retrospective studies with small sample sizes or individual case reports^2,5^. A consensus for the treatment of ESOS is not currently well defined.

The outcomes of classic osteosarcoma have been significantly improved owing to comprehensive treatment regimens based on both surgery and combined chemotherapy^6^. However, the role of chemotherapy on the outcomes of ESOS is still at debate. Recent evidence shows that it is still unclear that whether surgical treatment combined with chemotherapy could benefit the survival of patients with ESOS^2,7,8^. Therefore, in this study, we performed a retrospective analysis of the cases of 22 patients with ESOS, who received treatment in our hospital from January 1990 to June 2016, aiming to evaluate the postoperative outcomes of ESOS, and to predict the potential prognosis factors in ESOS.

## Material and Methods

### Patients

From January 1990 to June 2016, a total of 22 patients with ESOS were enrolled who underwent surgery as an initial treatment at Tianjin Cancer Hospital. The ESOS diagnosis was confirmed by pathologic examination after resection. In reference to other similar studies^9,10^, the inclusion criteria in this work were defined as follows: (1) Patients underwent ESOS resection with no previous history of skeletal osteosarcoma; (2) The tumor was located outside the solid organ (e.g., breast, prostate, etc.); (3) Patients did not receive previous regional radiotherapy. All patients were observed starting at 1 month after completion of treatment, then every 3 months for the first year, and every 6 months thereafter.

Tumor recurrence and metastasis were determined by imaging data (CT, MRI, or PET-CT) combined with tumor markers. The maximum diameter of the tumor was used as a scalar measure of tumor size. The tumor location was summarized as trunk (chest wall, pelvis, perineum, and head and neck) or extremity (upper and lower extremity, including shoulder, hip, and groin). Tumor depth is bounded by investing fascia: Deep is defined as in deep investing fascia, and Superficial is found in investing fascia. Margin status at initial resection was defined as follows: R0 for microscopically negative resections, R1 for microscopically positive resections and R2 for macroscopically positive resections, respectively. The Federation Nationale des Centers de Lutte Contre le Cancer (FNFLFF) grading system was used for tumor grading^11^. Grade I-II tumors were considered as low grade, whereas Grade III-IV tumors were considered high grade. Local recurrence-free survival (LRFS) was defined as the time from resection to local relapse. Overall survival (OS) time was measured from the date of resection to the date of death or the last follow-up visit. Progression-free survival (PFS) was defined as the time between the date of resection and the objective tumor progression or death. The response of tumors to preoperative therapy was evaluated using the RECIST1.1^12^.

### Statistical analysis

SPSS software (version 19.0) was used for Statistical analysis. Kaplan Meier method was applied to analyze the local recurrence-free survival (LRFS), PFS, and OS rate. Univariate analysis was employed to evaluate potential risk factors such as tumor size, pathological grade, tumor depth, stage, surgical resection, and adjuvant treatment types. Log-rank test was used for comparison. P values of less than 0.05 were considered statistically significant.

### Ethical approval

This study was approved by Local Ethics Committee of Tianjin Medical University Cancer Institute & Hospital in compliance with the Helsinki Declaration, and informed consent was obtained from all participants. The patient records were anonymized and de-identified before analysis to protect the privacy of patients and to safeguard the confidentiality of patient health and other private information. This article does not contain any studies with animals performed by any of the authors.

## Results

### Characteristics of Patients, tumor, and treatment

A total of 22 patients (15 males and 7 females) diagnosed with ESOS were enrolled, with a median age of 55.5 years. 19 patients suffered with localized disease and 3 presented with synchronous metastasis in lung at diagnosis. The primary tumor sites were reported as follows: 3 in the chest wall, 2 in the latissimus dorsi muscle, 1 in the erector spine, 1 in the retroperitoneum, 2 in the upper extremity and 13 in the lower extremity. The median tumor size was 5.9 cm and the mean tumor size was 5.5 cm (range, 1.7–15.1 cm).

All patients received resections for primary diseases. The surgical procedures performed consisted of 2 (9.1%) cases of local resection, 17 (77.3%) cases of wide excision and 3 (13.6%) cases of amputation. 7 (31.8%) primary lesions were found in superficial layer and 15 (68.2%) ones were located in deep layer. After surgery, 18 (81.8%) patients had negative margins (R0), while 4 (18.2%) patients had microscopically positive margins (R1). For these 4 patients with R1 resection, two patients refused the radiotherapy and only 2 cases with R1 resection (lesions located in retroperineum and leg) received radiotherapy after the surgery. One patient with retroperineum lesion after R1 resection suffered from recurrence after the radiotherapy 11 month later. The other patient with leg lesion after R1 resection suffered from metastasis after 12 months but had no recurrence after 17 months’ follow-up. 17 (77.3%) patients had high-grade malignancy and 5 (22.7%) patients were of low grade tumor. 18 (81.8%) patients had received combination chemotherapy, including 15 cases of adjuvant chemotherapy, and 3 cases of neoadjuvant chemotherapy plus adjuvant chemotherapy, leaving 4 (18.2%) cases receiving no chemotherapy. The chemotherapy regimens were methotrexate, anthracycline, cisplatin and ifosfamide (MAP plus ifosfamide). Two cycles each of high-dose methotrexate (10–12 g/m^2^), doxorubicin (75 mg/m^2^) and cisplatin (120 mg/m^2^) were given as neoadjuvant chemotherapy. Postoperatively, patients received three cycles of methotrexate, ifosfamide (15 g/m^2^) and doxorubicin/cisplatin. The characteristics of the patients, tumor, and treatment were summarized in Table 1.

**Table 1:** Characteristics of 22 ESOS patients.

Characteristics	Value n (%)
No. of patients	22 (100%)
Ages (years) median (range)	55.5 (21–72)
**Sex**	
female	7 (32%)
male	15 (68%)
**TNM stage**	
IIA	5 (23%)
IIB	14 (64%)
IV	3 (14%)
Tumor diameter (cm) median (range)	5.9 (3.1–15.1)
mean (range)	5.5 (3.1–15.1)
**Histologic Grade**	
low	5 (23%)
high	17 (77%)
**Tumor location**	
trunk	7 (32%)
extremity	15 (68%)
**Tumor depth**	
superficial	7 (32%)
deep	15 (68%)
**Operative type**	
local	2 (9%)
wide	17 (77%)
amputation	3 (14%)
**Surgery margin status**	
R0	18 (82%)
R1	4 (18%)
**Chemotherapy**	
Yes	18 (82%)
adjuvant	15 (68%)
neoadjuvant + adjuvant	3 (14%)
None	4 (18%)
**Radiotherapy**	
Yes	2 (9%)
No	20 (91%)

### Recurrence and metastases

**Figure 1:**
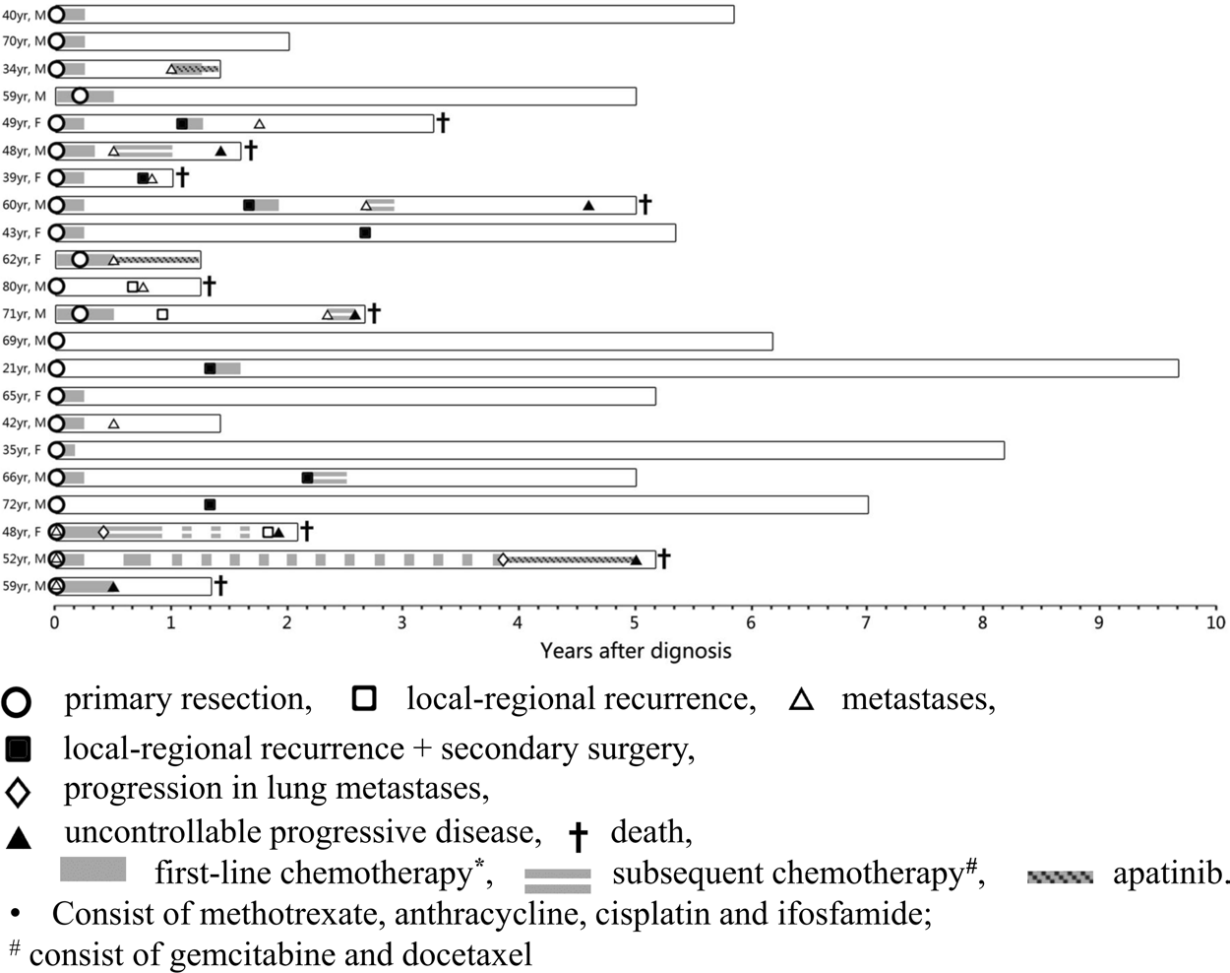
Details of 22 patients’ clinical courses.

Local-regional recurrences were experienced by 10 of 22 (45.5%) patients. Secondary surgery was performed for 7 (31.8%) patients. Of patients with localized diseases (N = 19), 9 (47.3%) patients developed distant metastases. Salvage chemotherapy was used in 7 patients after treatment failure. The clinical courses and the timings of all cases were summarized in Fig. 1.

**Figure 2:**
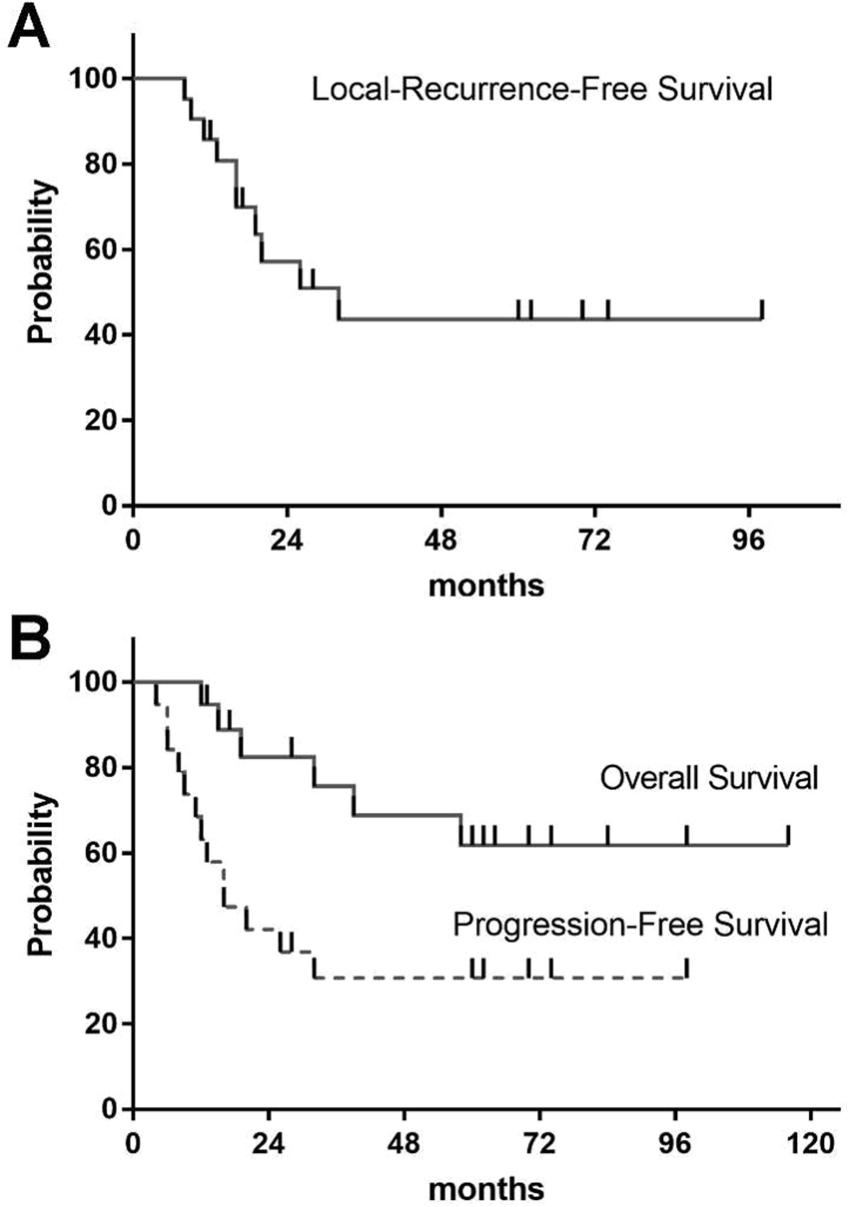
Survival rate of ESOS patients. (**A**) Local recurrence free survival for all patients (N = 22). (**B**) Kaplan-Meier estimates for overall survival and progression-free survival in patients with localized diseases (N = 19).

For all patients, the 3-year local-recurrence-free survival (LRFS) rate was 44% with a median LRFS time of 32 months (Fig. 2A). For localized cohort, a total of 13 (63.2%)patients developed disease progression. The median progression-free survival time (PFS) was 16 months with a 3-year PFS rate of 31% (Fig. 2B). Only the tumor diameter factor was statistically significant prognostic factor of PFS (Table 2, *p* = 0.008) in the univariate analysis. Low histologic grade and negative margin status was also associated with better PFS, although the difference was not statistically significant (Table 2, *p* = 0.081 and *p* = 0.075, respectively). Combination (adjuvant and/or neo-adjuvant) chemotherapy was given in 15 of the 19 localized lesion patients, but the receipt of chemotherapy did not significantly affect the OS (*p* = 0.638) or PFS (*p* = 0.665) outcomes. However, this is difficult to interpret due to the low number of patients without chemotherapy.

**Table 2:** Univariate analysis for overall survival and progression-free survival in localized cohort (N = 19).

Variables	No. of patients	Overall Survival		Progression-Free Survival	
		3 y OS rate (%)	P value	3 y PFS rate (%)	P value
**Ages (y)**			0.748		0.431
≤56	9	74.1		22.2	
>56	10	76.2		40	
**Sex**			0.893		0.905
female	6	62.5		30.8	
male	13	72.2		33.3	
**Tumor diameter (cm)**			0.017		0.008
≤5.5	9	87.5		53.3	
>5.5	10	46.3		10	
**Histologic grade**			0.079		0.081
low	5	100		53.3	
high	14	65.7		21.4	
**Tumor location**			0.513		0.737
trunk	6	66.7		33.3	
extremity	13	82.1		28.8	
**Tumor depth**			0.038		0.383
superficial	6	100		25	
deep	13	62.3		30.8	
**TNM stage**			0.079		0.081
IIA	5	100		53.3	
IIB	14	65.7		21.4	
**Operative type**			0.296		0.153
local	2	0		50	
wide	17	73.3		34.3	
**Margin status**			0.022		0.075
R0	15	77		38.9	
R1	4	37.5		0	
**Combination CT**			0.638		0.665
without	4	75		40	
with	15	75.4		28.6	

### Overall survival

Patients were followed-up until death or June 1, 2016. The median follow-up time for all patients was 48.5 months. At the time of analysis, a total of 9 (40.9%) patients had died and each died of cancer. The 3-year and 5-year OS rate for all patients were 69% and 58%, respectively (Fig. 2B). For those who experienced localized diseases (N = 19), the 3-year and 5-year OS were 76% and 62%, respectively (Fig. 2B). On univariate analysis, parameters such as positive margin (*p* = 0.022), deep tumor location (*p* = 0.038) and tumor mean diameter ≥5.5 cm *(p* = 0.017) were associated with worse survival status (Table 2).

### Typical case

**Figure 3:**
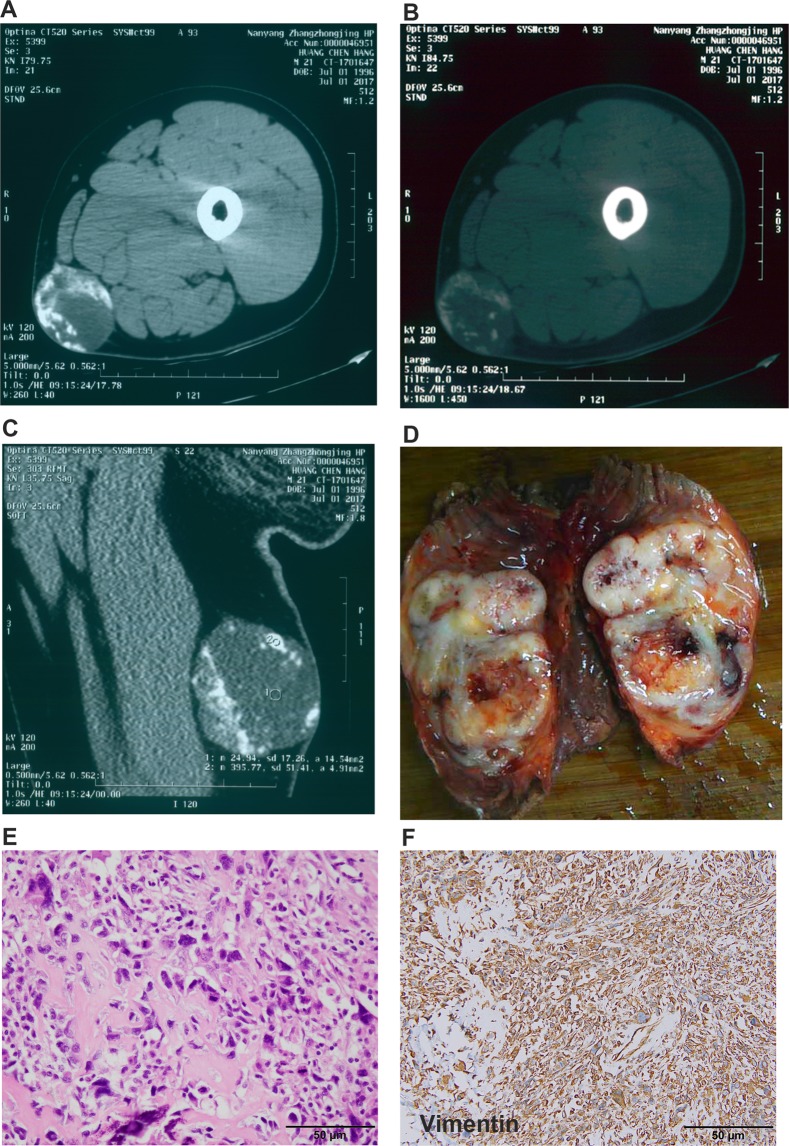
Typical leg ESOS. (**A–C**) CT scan showed big mass with calcification. (**D–F**) The pathological diagnosis was confirmed the ESOS.

Male patient (40 years old) was suffered from pain and mass in the leg (Fig. 3). CT scan showed big mass with calcification (Fig. 3A–C). Needle biopsy was performed and the pathological diagnosis suggested ESOS. Wide resection was performed and acquired RO margin. The pathological diagnosis was confirmed the ESOS (Fig. D–F**)**. Adjuvant chemotherapy after surgery included MTX + IFO + DDP + ADM for 3 cycles. No recurrence and metastasis were detected after 70 months’ follow-up. Also, published data about ESOS were also summarized and listed (Table 3).

**Table 3:** Literature review of surgery outcomes for ESOS.

Reference	Year	Cases (metastases)	Median Age	OS rate	PFS rate (localized)
Lee JS^13^	1995	40 (0)	50.7 (mean)	5 y 37%	—
Ahmad SA^15^	2002	60 (22)	55	5 y 46% (30 pts)	5 y 46%
Torigoe T^16^	2007	20 (1)	50	5 y 66%	—
Choi LE^9^	2014	53 (11)	64	3 y 61%	3 y 50%
Thampi S^10^	2014	256 (68)	60.7 (mean)	5 y 37% (47% for localized)	—
Berner K^17^	2015	37 (8)*	68	5 y 16%	—
Fan Z^8^	2015	36 (0)	59 (mean)	5 y 53%	—
Longhi A^14^	2017	266 (55)	57	5 y 47% (51.4% for localized)	5 y 43%
Paludo J^18^	2017	43 (6)	55	5 y 45%	5 y 44%
Current study	2018	22 (3)	55.5	5 y 58%	3 y 31%

*OS* Overall survival; PFS progression-free-survival.

*Only 29 pts received surgery in Berner’s investigation.

## Discussion

ESOS is a rare malignant subtype of osteosarcoma, sharing histological features with primary bone osteosarcoma but without attachment to bone or periosteum^1–4^. The exact etiology of ESOS is still unknown but several associated prognostic factors have been proposed, such as the history of trauma, local radiation therapy, and changes in soft tissue lesions and malignant fibrous tissue disease, and understanding the etiology of this disease demands greater understanding of the cell of origin of ESOS. To date, there is still a lack of specific clinical manifestations of the disease, mostly showing limb pain and swelling of the affected area, some patients may have fatigue, anorexia, body weight loss and other systemic symptoms^2–5^. Different from classical OS, ESOS has a higher age of onset, and the peak age of onset is more than 50 years old^2^. The median age of this study cohort was 55.5 years, which was in line with the median age of 50–60 years reported in previous literature^2^. Besides, previous studies indicated that this disease occurs slightly more common in males than in females, however, a few recent reports observed female predominance for ESOS, suggesting the preconceived gender distribution may be not necessarily the actual case^9,10^. In addition, in terms of tumor sites, ESOS most often affects the lower limbs, followed by the upper limbs^2,4^. Consistent with prior report, our study found that 59% of patients had tumors located in low extremity.

Constrained by the extremely low incidence of ESOS, there is still a paucity of research on this disease. Its treatment plan still largely refers to the standard treatment plan for classic osteosarcoma. In 1995, Lee JS *et al*.^13^ first reported the postoperative efficacy of ESOS. The study included 40 cases of ESOS patients with a mean age of 50.7 years who were treated surgically. The results showed that all 40 ESOS patients had a 37% 5-year OS rate. Thampi S^10^ and Longhi A^14^ conducted two multi-centre studies, exploring outcomes of 256 and 266 ESOS patients respectively. For patients with localized diseases, the former obtained a 5-year OS rate of 47%, while the later showed 5-year OS and 5-year PFS rates of 51.4% and 43%, respectively. Previous studies are mostly multi-center retrospective studies. The gap between the time of their sample enrollment is quite large, which often spans more than 30 years, and there are few studies with large enough samples of evidence that are credible. Collectively, those situations have led to the present paucity of evidence-based proof for thorough understanding of ESOS. Its disease characteristics have not been fully explored, and its treatment depends largely on standard treatment options for classical osteosarcoma.

We have summarized the outcomes of ESOS after surgical treatment and chemotherapy in both prior and our present studies^9,10,13–18^. In general, the 5-year OS rate ranged between 37% and 51%, the 5-year PFS rate was about 45%. Most studies have indicated higher patient age, larger tumor volume, and tumor distribution in the vertical axis as poor survival factors. In the present study, we retrospectively analyzed 22 cases of ESOS patients who underwent surgical resection in the center from 1990 to 2016. Survival analysis (Fig. 1 and Table 2) showed 62% of 5-year OS rate and 33% of 5-year PFS rate for the localized diseases, which were similar to other studies. Furthermore, our study showed that the tumor diameter of greater than 5.5 cm and high-grade pathological grade were poor prognostic factors for ESOS (Table 1). The problem of local recurrence after surgery is one of the difficulties in the treatment of ESOS. Previous studies have reported a 45–50% local recurrence rate. Similar to other reports^13–15^, we noticed 10 cases of local recurrence accounting for a recurrence rate of 45.5%, and all patients relapsed within 3 years after surgery. It is really higher than typical osteosarcoma. However, it still does not know whether the higher recurrent rate is due to the less radiotherapy after surgery or their extraskeletal locations. This might suggest that increasing the safe resection margin might be necessary for ESOS treatment and also more investigations are needed to make this question clear.

The efficacy and benefit of chemotherapy on ESOS is controversial. In the early explorations, Patel have examined the efficacy of ifosfamide and gemcitabine in bone and soft tissue sarcomas, and results indicated positive dose-responsive therapeutic effects^19,20^. Bramwell *et al*.^21^ used a single-agent doxorubicin-based chemotherapy regimen for soft tissue sarcoma and found that compared to single-agent doxorubicin, combination chemotherapy produced only marginal increase in response rates and no improvements in overall survival. In Ahmad’s study, 27 patients received adriamycin-based chemotherapy. The total effective rate of chemotherapy was only 19%, but no specific effect of chemotherapy on survival was given^15^. In 2005, Goldstein *et al*.^22^ reported that combination of multiagent chemotherapy with surgery produced a surprisingly good survival rate in 17 eligible ESOS patients. In 2007, a Japanese^16^ study reported 20 patients with ESOS. Most of the cohort (15/20, 75%) received doxorubicin and/or platinum chemotherapy, resulting a partial response rate of 33%. For the case without chemotherapy, the OS rate was reduced by 25%. However, in 2014 Choi LE^9^ reported that no survival improvement was found in the treatment of doxorubicin-based chemotherapy. In 2017, A European study with 266 patients further demonstrated that compared with doxorubicin-based chemotherapy, platinum-based classical osteosarcoma chemotherapy is more beneficial to patients’ long-term prognosis^14^. Paludo’s study exported a same trend in the next year^18^. Though Fan *et al*. suggested that multimodality treatment that includes doxorubicin and ifosfamide-based chemotherapy, radiation, and surgery may be a valid therapeutic strategy for stage III ESOS^8^, the utilization of chemotherapy, especially for high-grade ESOS, remains a subject of further exploration. In our study, chemotherapy based on MAP plus ifosfamide did not show any significant survival benefit, either in the limited period or in all patients. Considering the small sample size of present studies, more large-scale studies are needed to further elucidate the role of chemotherapy in Asian ESOS population.

The patients enrolled in this study had a large span of time period, and there was certain heterogeneity within the samples. Furthermore, owing to the small sample size, we cannot properly analyze the prognostic impact of treatment factors such as chemotherapy. However, as a single-center study of ESOS, this study had a smaller bias in treatment strategy compared to previous multicenter studies. Furthermore, to our best knowledge, only one study for Asian population is available^16^. As one of the few ESOS experiences in Asians, this study provides a new evidence-based groundwork for the diagnosis and treatment of ESOS and has certain reference values.

In all, the present study with largest Chinese cohort reveals that larger tumor size and higher histologic grade in ESOS patients indicate a lower OS rate. In addition, this study demonstrates that the combination chemotherapy based on MAP plus ifosfamide does not improve the OS or PFS rate. Due to the extremely low incidence of this disease and as prospective studies are difficult to achieve, multicenter retrospective studies with large sample sizes may be a good way to further explore the optimal treatment of ESOS, especially for Asian population.

## Data Availability

The datasets generated during and/or analyzed during the current study are available from the corresponding author upon reasonable requests.
